# Supplementary material for: Does reef crest zone selection influence Acropora palmata (Lamarck, 1816) fragment survival and growth?
Source: PeerJ. 2025 Nov 14;13:e20303. doi: 10.7717/peerj.20303 (PMC12622234; doi:10.7717/peerj.20303)
Supplement: Supplemental Information 2 [file peerj-13-20303-s002.docx]

Analysis scripts

- A Cox regression model, was used to analyze whether the initial size of the fragments influenced survival, using the survival package (Therneau & Grambsch, 2000).

library(survival)

cox_model <- coxph(Surv(Time, Censored) ~ height + width, data = Data)

summary (cox_model)

- The survival probability (sp) of the fragments was evaluated using the nonparametric Kaplan-Meier estimator with the survminer package (Kassambara et al., 2021).

library(survminer)

library(ggpubr)

library(ggplot2)

library(dplyr)

survival probability <-survfit (Surv( Time, Censored) ~ Zone, data = Data,

type= "kaplan-meier")

summary (survival probability)

ggsurvplot(survival probability, pval = TRUE, xlab = "Time", ylab = "Survival")

- A generalized and linear mixed model was used to estimate the effect of: (1) the water flow represented by the dissolution of the plaster discs, (2) mean temperature in each period, (3) depth, (4) period, (5) crest zone and (6) crest site on fragment survival and growth rate, respectively with the lme4 package (Bates et al., 2015).

<- glmer ( survival ~ dissolution + temperature + depth + period + crest * zone + (1 | id_colonie), data = Data, family = binomial)

<- lmer (growth ~ dissolution + Temperature * period + crest * zone + (1 | id_colonie), Data= Data)

*glmer → survival (binary response, binomial distribution).*

*lmer → growth (continuous response, normal distribution).*

- A segmented model was used to identify dissolution values from which the effect on growth begins to change using the segmented package (Muggeo, 2008).

library(segmented)

mod_base <- lm(growth ~ dissolution, data = Data)

mod_seg <- segmented(mod_base, seg.Z = ~ dissolution)

summary(mod_seg)

plot(mod_seg)
