# Supplementary material for: Does reef crest zone selection influence Acropora palmata (Lamarck, 1816) fragment survival and growth?
Source: PeerJ. 2025 Nov 14;13:e20303. doi: 10.7717/peerj.20303 (PMC12622234; doi:10.7717/peerj.20303)
Supplement: Supplemental Information 3 — The p-value ≤ 0.05 indicates significant differences between crest zones. [file peerj-13-20303-s003.docx]

Table S1 Initial mean size (± Standard deviation (SD)) of *A. palmata* fragments in the fore and back zones on the crests at Playa Baracoa (PB), Rincón de Guanabo (RG), El Peruano (Pr) and Mariflores (Mr). The p-value ≤ 0.05 indicates significant differences between crest zones.

| **Site** | **Zone** | **Width (cm) (mean ± SD)** | **Height (cm) (mean ± SD)** |
| --- | --- | --- | --- |
| PB | Fore | 3.2 ±1.1 | 3.9 ± 1 |
|  | Back | 3.1 ± 0.9 | 3.7 ± 1.3 |
| p-value |  | T = -0.1 df = 40.5, p = 0.9 | T = 0.8, df = 38.1, p = 0.4 |
| RG | Fore | 2.6 ± 0.8 | 3.4 ± 0.9 |
|  | Back | 2.7 ± 0.7 | 3.1 ± 1 |
| p-value |  | T = -0.1, df = 31.9, p = 0.9 | t = 0. 8, df = 28.5, p = 0.4 |
| Pr | Fore | 2.7 ± 0.8 | 2.9 ± 1 |
|  | Back | 2.9 ± 0.8 | 2.8 ± 0.7 |
| p-value |  | t = 0.6, df = 35.9, p = 0.5 | t = -0.09, df = 31.5, p = 0.9 |
| Mf | Fore | 2.3 ± 0.5 | 2.4 ± 0.9 |
|  | Back | 1.9 ± 0.4 | 2.4 ± 0.6 |
| p-value |  | T = -3.2, df = 41.4, **p =** **0.003** | W = 262.5, p = 0.9 |
