# Supplementary material for: Does reef crest zone selection influence Acropora palmata (Lamarck, 1816) fragment survival and growth?
Source: PeerJ. 2025 Nov 14;13:e20303. doi: 10.7717/peerj.20303 (PMC12622234; doi:10.7717/peerj.20303)
Supplement: Supplemental Information 4 — The p-value ≤ 0.05 indicates significant differences between crests. [file peerj-13-20303-s004.docx]

Table S2 Significance value (p ≤ 0.05) of non-parametric Kruskal-Wallis (K-W) and Dunn tests for initial sizes (width and height) of *A. palmata* fragments collected at Playa Baracoa (PB), Rincón de Guanabo (RG) El Peruano (Pr) and Mariflores (Mf) crests from the fore and back zones. The p-value ≤ 0.05 indicates significant differences between crests.

|  | **p- value** |  |
| --- | --- | --- |
| **Zone** | **Width** | **Height** |
| Fore | X^2^ = 8.2, p = 0.04  PB-Mf, p = 0.02 | X^2^ = 16.2, p = 0.001  PB-Mf, p = 0.0003  PB-Pr, p = 0.03 |
| Back | X^2^ = 26.1, p = 0.001  PB-Mf, p = 0.001  Pr-Mf, p = 0.0002  RG-Mf, p = 0.009 | X^2^ = 23.3, p = 0.001  PB-Mf, p = 0.001  RG-Mf, p = 0.003 |
