# Supplementary material for: Does reef crest zone selection influence Acropora palmata (Lamarck, 1816) fragment survival and growth?
Source: PeerJ. 2025 Nov 14;13:e20303. doi: 10.7717/peerj.20303 (PMC12622234; doi:10.7717/peerj.20303)
Supplement: Supplemental Information 5 — The p value ≤ 0.05 (t-Student) indicates significant differences between zones, as indicated in hold. N-value represents the number of total fragments measured for each time. When the value is negative, there was a decrease in growth rate. [file peerj-13-20303-s005.docx]

Table S3 The growth rate (cm year^-1^ in width and height) (± standard deviation, SD) of *Acropora palmata* fragments located in the fore and back crest zones for each study period in Playa Baracoa (PB), Rincon de Guanabo (RG), El Peruano (Pr) and Mariflores (Mf). The p value ≤ 0.05 (t-Student) indicates significant differences between zones, as indicated in hold. N-value represents the number of total fragments measured for each time. When the value is negative, there was a decrease in growth rate.

| **Reef** | **Time**  **(days)** | **Zone** | **N** | **Width**  **(cm year^-1^)** | **p** | **Height**  **(cm year^-1^)** | **p** |
| --- | --- | --- | --- | --- | --- | --- | --- |
|  | 152 | Fore | 23 | 1.8 ± 1.5 | **0.006** | 0.7 ± 2.9 | 0.3 |
|  |  | Back | 20 | 0.4 ± 1.5 |  | 1.8 ± 3.7 |  |
|  | 279 | Fore | 22 | .7 ± 2.2 | 0.06 | 3.7 ± 3.7 | 0.1 |
| PB |  | Back | 15 | 2.2 ± 2.2 |  | 1.8 ± 2.2 |  |
|  | 453 | Fore | 18 | -0.1 ± 2.6 | **0.02** | 0.7 ± 2.2 | **0.03** |
|  |  | Back | 11 | 1.8 ± 1.8 |  | 2.9 ± 2.9 |  |
|  | 136 | Fore | 23 | 1.1 ± 1.5 | 0.2 | 1.5 ± 2.6 | 0.7 |
|  |  | Back | 16 | 0.7 ± 1.1 |  | 1.1 ± 2.2 |  |
| RG | 261 | Fore | 19 | 1.8 ± 1.8 | 0.06 | 2.9 ± 3.7 | 0.5 |
|  |  | Back | 8 | 7.3 ± 3.7 |  | 3.7 ± 3.7 |  |
|  | 433 | Fore | 18 | 0.3 ± 2.6 | 0.3 | 0.7 ± 2.6 | **0.02** |
|  |  | Back | 6 | 2.2 ± 3.7 |  | 3.7 ± 2.2 |  |
|  | 172 | Fore | 18 | 0.4 ± 2.2 | **0.03** | 1.8 ± 2.9 | 0.3 |
|  |  | Back | 18 | 2.2 ± 1.8 |  | 2.6 ± 2.2 |  |
| Pr | 291 | Fore | 12 | 3.7 ± 3.7 | **0.02** | 1.1 ± 2.6 | 0.5 |
|  |  | Back | 16 | 0.2 ± 2.2 |  | 0.3 ± 2.9 |  |
|  | 423 | Fore | 12 | 2.9 ± 2.2 | 0.9 | 2.2 ± 2.9 | **0.04** |
|  |  | Back | 17 | 2.9 ± 3.7 |  | 3.7 ± 3.7 |  |
|  | 168 | Fore | 18 | 1.1 ± 2.6 | **0.03** | 2.2 ± 2.9 | 0.7 |
|  |  | Back | 17 | 3.3 ± 2.6 |  | 2.6 ± 3.3 |  |
| Mf | 291 | Fore | 19 | -0.4 ± 2.6 | **0.0002** | -1.1 ± 2.6 | **0.001** |
|  |  | Back | 16 | 2.6 ± 1.8 |  | 2.6 ± 1.8 |  |
|  | 423 | Fore | 16 | 0.1 ± 1.5 | 0.09 | 1.5 ± 2.2 | 0.3 |
|  |  | Back | 14 | -14.6 ± 2.6 |  | 0.7 ± 1.8 |  |
